# 42 Motives and Barriers of Active Transportation as a Sustainable Physical Activity among Young Lithuanian Adults

**DOI:** 10.1093/eurpub/ckae114.088

**Published:** 2024-09-26

**Authors:** Brigita Mieziene, Laima Gasiuniene

**Affiliations:** Lithuanian Sports University, Kaunas, Lithuania; Lithuanian Sports University, Kaunas, Lithuania

## Abstract

**Purpose:**

Active transportation (AT) as a sustainable form of physical activity that contributes to both personal health and the environment is of the utmost importance. However, the prevalence of AT and its related predictors are still understudied. So, this study aims to identify motives and barriers for AT among the Lithuanian adult population. The study seeks to answer what drives people to use AT and what prevents them from using it.

**Methods:**

The cross-sectional study was conducted that included 250 Lithuanian young adults aged 18-39 years, the mean age was 20.38 years. Among them 80% were women. AT was measured using the Stages of Change model-based questionnaire. Mean comparison and logistic regressions were performed to identify significant predictors of AT. Motives were measeured following Kim & Hall (2022). Barriers assessed with questions developed by Hoj et al. (2018).

**Results:**

Results indicated that 27.6% of young people use active transportation. AT was predicted by higher scores on the Motives scale (Expβ=1.41 [1.10-1.81], p = 0.007) and lower on the Barriers scale (Expβ=0.76 [0.68-3.58], p = 0.018). Among particular motives, there were significant differences between using and not using AT of the following: physical health, emotional health, relaxation, and opportunity to be in nature with higher scores of those using AT. Also, practical motives to get to the destination, avoid traffic jams, and not own a car. However, saving the environment was not significantly related to using AT. Among barriers, time restrictions, several destinations to reach, inconvenient landscapes, and troubles with outfits were more prominent among those not using AT.

**Conclusions:**

Young people engage in AT mostly because of their motives and avoid engaging because of practical barriers. Awareness of the importance of individual AT for the welfare of the environment should be promoted and further studied in experimental studies.

